# Kidney function and cognitive impairment

**DOI:** 10.1113/EP091003

**Published:** 2022-12-20

**Authors:** Matthew A. Bailey

**Affiliations:** ^1^ Edinburgh Kidney, British Heart Foundation Centre for Cardiovascular Science University of Edinburgh Edinburgh UK

**Keywords:** chronic kidney disease, cognition, dementia

1

A persistent decline in glomerular filtration rate is diagnostic of chronic kidney disease (CKD), a condition affecting ∼800 million people world‐wide and incurring high costs to healthcare providers. That CKD substantially increases cardiovascular risk is well known, but it is also evident that living kidney donation has a negative long‐term impact on the cardiovascular system. Less widely appreciated is the detrimental effect that kidney disease has on brain health. CKD is a strong risk factor for developing dementia (Viggiano et al., 2020), and mild cognitive impairment, such as a decline in attention and short‐term memory, may be evident even in the early stages of kidney disease (Brodski et al., 2019). The nature of this relationship between kidney function and cognition is largely unknown but it is clearly complex, since cognitive impairment can persist in those who have received a kidney transplant (Ziengs et al., 2022).

In this issue of *Experimental Physiology*, Basta et al. (2023) use rats to model living kidney donation by surgically removing the right kidney under anaesthesia. Twenty weeks later, the rats performed a Morris water maze test and a T‐maze spontaneous alternation test to evaluate spatial memory. Compared with un‐operated control rats, animals with only a single functional kidney showed impaired performance in both tasks. Post‐mortem examination of the brain found evidence of oxidative stress in the hippocampus of uni‐nephrectomised rats. These animals also had a higher number of hippocampal cells expressing caspase‐3 than control animals, indicative of activation of the apoptotic pathway for programmed cell death. More hippocampal cells expressed glial fibrillary acidic protein, suggesting astrocytic activation, and expression of brain‐derived neurotrophic factor was reduced. Overall, the findings of this study suggest that removal of a kidney promotes gliosis and/or other neural damage, which may account for the poor performance of the uni‐nephrectomised rats in tests of spatial memory.

An intriguing aspect of the study was the inclusion of an interventional group, in which rats received a daily oral supplement of zinc sulphate, starting 1 week after the removal of the kidney and continuing until the end of the experiment at 20 weeks. Zinc is an essential trace metal with antioxidant properties (Powell, 2000) and is commonly deficient in patients with kidney disease. Zinc supplements have beneficial cardiovascular effects in CKD (Nakatani et al., 2021), and in the brain, where most zinc is protein bound, ‘free zinc’ is released from synaptic vesicles to act as a postsynaptic neuromodulator at glutamatergic neurons. In the study by Basta et al. (2023), the zinc sulphate supplement strategy partially prevented the zinc depletion that occurred following removal of a kidney. In the spatial learning tasks, the performance of the zinc‐treated animals was not different from that of the control group, and within the hippocampus signs of oxidative stress, apoptosis and neural injury were largely ameliorated.

The impact of kidney donation on brain health has not been extensively investigated, but what data there are suggest that cognitive function may be impaired to some degree (Mikuteit et al., 2022). The work by Basta et al. (2023) suggests that the extent of cognitive decline post‐donation is likely to be determined by the functionality of the remaining kidney. In their study, removal of one kidney induced oxidative stress, fibrosis and glomerular remodelling in the remaining organ, along with metabolic abnormalities of hyperglycaemia and dyslipidaemia. These renal and systemic abnormalities were all improved in rats receiving oral zinc sulphate supplements.

Extracellular zinc is intrinsically neurotoxic, and zinc‐based therapeutic approaches for neurological conditions are therefore controversial (Levenson, 2005). There is, nevertheless, a pressing need for new approaches to the treatment of dementia. The translational implications of the study by Basta et al. (2023) are fascinating and the study provides an excellent foundation for improving our mechanistic understanding of the complex interplay between kidney physiology and cognitive function.

## AUTHOR CONTRIBUTIONS

Sole author.

## CONFLICT OF INTEREST

None.

## FUNDING INFORMATION

No funding was received for this work.
